# Ketogenic diet-mediated steroid metabolism reprogramming improves the immune microenvironment and myelin growth in spinal cord injury rats according to gene and co-expression network analyses

**DOI:** 10.18632/aging.202969

**Published:** 2021-05-06

**Authors:** Hong Zeng, Yao Lu, Meng-Jie Huang, Yan-Yan Yang, Hua-Yi Xing, Xiao-Xie Liu, Mou-Wang Zhou

**Affiliations:** 1 Department of Rehabilitation Medicine, Peking University Third Hospital, Beijing 100191, China; 2 Department of Rehabilitation Medicine, Shanghai Ninth People's Hospital Affiliated to Shanghai Jiao Tong University School of Medicine, Huangpu 200011, China

**Keywords:** spinal cord injury, ketogenic diet, RNA-seq, steroid biosynthesis, metabolic reprogramming, immune microenvironment

## Abstract

The ketogenic diet has been widely used in the treatment of various nervous system and metabolic-related diseases. Our previous research found that a ketogenic diet exerts a protective effect and promotes functional recovery after spinal cord injury. However, the mechanism of action is still unclear. In this study, different dietary feeding methods were used, and myelin expression and gene level changes were detected among different groups. We established 15 RNA-seq cDNA libraries from among 4 different groups. First, KEGG pathway enrichment of upregulated differentially expressed genes and gene set enrichment analysis of the ketogenic diet and normal diet groups indicated that a ketogenic diet significantly improved the steroid anabolic pathway in rats with spinal cord injury. Through cluster analysis, protein-protein interaction analysis and visualization of iPath metabolic pathways, it was determined that Sqle, Sc5d, Cyp51, Dhcr24, Msmo1, Hsd17b7, and Fdft1 expression changed significantly. Second, through weighted gene co-expression network analysis showed that rats fed a ketogenic diet showed a significant reduction in the expression of genes involved in immune-related pathways, including those associated with immunity and infectious diseases. A ketogenic diet may improve the immune microenvironment and myelin growth in rats with spinal cord injury through reprogramming of steroid metabolism.

## INTRODUCTION

Spinal cord injury (SCI) is a devastating event that can lead to neurological deficits and motor dysfunction [1–2]. The dysfunction of motor and sensory functions after SCI is caused by two processes: primary injury and secondary injury. Primary injury results from irreversible mechanical damage to the spinal cord, which directly damages various tissue components. In contrast, secondary injury is a cascade of effects triggered by primary injury that presents as a variety of biochemical and pathological events, such as oedema, inflammation and apoptosis [3–4]. Current treatment strategies for SCI aim to protect neurons from secondary damage [5–6]. However, therapy is effective only in a limited scope [7]. Thus, understanding the molecular changes in SCI after therapy could lead to improved treatment of patients with SCI.

A ketogenic diet (KD) is a high-fat, low-carbohydrate, moderate-protein diet that results in the prominent production of ketone bodies (KBs: β-hydroxybutyrate [BHB], acetoacetate [ACA] and acetone) [8]. Furthermore, the KD has been validated as an effective non-pharmacological preventive treatment for drug-resistant epilepsy [9–10]. Interestingly, recent studies have suggested that a KD might also be beneficial to humans with neurological disorders, such as Parkinson’s disease, Alzheimer’s disease, multiple sclerosis, and traumatic brain injury [11–13]. Multiple reports have revealed that a KD may exert neuroprotective effects after SCI [14–19]. For example, Tetzlaff et al. found that KD improves forelimb motor function after SCI [14]. Zhu et al. revealed that a KD exerts neuroprotective effects by attenuating oxidative stress in SCI rats [18–19]. Finally, our study recently reported that a KD improves motor function by activating Nrf2 and suppressing the NF-κB signalling pathway to attenuate oxidative stress and inflammation after SCI [17]. However, the underlying mechanisms by which a KD provides neuroprotection after SCI are still not fully understood.

Based on the above information, in this study, we performed RNA sequencing (RNA-seq) to examine the changes in the gene expression of spinal cord tissue in Sprague-Dawley rats under with different conditions. We identified differentially expressed genes (DEGs) and comprehensively detected genes with important biological significance through gene set enrichment analysis (GSEA) at the overall level [20]. Weighted gene co-expression network analysis (WGCNA) was used to establish a gene co-expression network to explore the relationship between the gene network and the KD diet phenotype [21]. These data promote further understanding of the new genes and related signalling pathways and how KD treatment improves the recovery of motor function in SCI rats, providing new guidance for clinical treatment.

## RESULTS

### Experimental procedures and animal conditions

**Figure 1:**
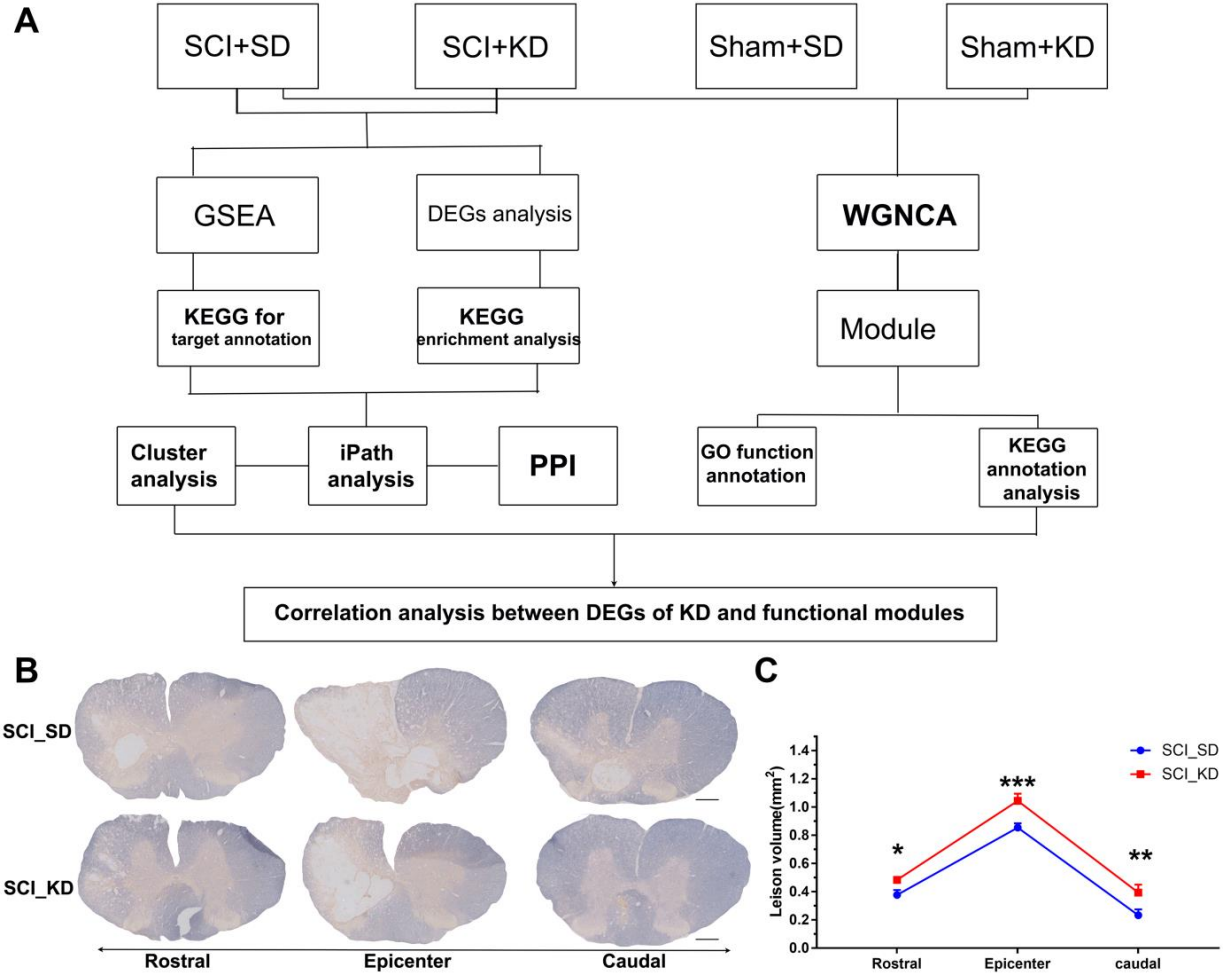
**Experimental protocol and animal myelin area.** (**A**) Detailed experimental protocol. (**B**) Representative images showing EC staining after SCI. Scale bar = 1 mm. (**C**) Quantitative analysis of the results in panel (A). All data are presented as the mean ± standard deviation, *n* = 3. Rostral of the injury site, *p* = 0.0255; Epicentre, *p* = 0.0004; Caudal, *p* = 0.0015.

The entire experimental process is shown in Figure 1A. To establish a C7 spinal cord hemi-contusion model, unilateral C7 laminectomies were performed using a Precision Systems and Instrumentation-IH Impactor according to the manufacturer’s instructions (Supplementary Figure 1A). Sprague-Dawley rats were anaesthetized with sodium pentobarbital to expose the spinal cord. We fixed the C6-T1 vertebrae rigidly in a frame tilted at a 25.0° angle and then used the Infinite Horizon impactor to deliver a set contusion force of 150 kdyne (Supplementary Figure 1B); the contusion forces were monitored by a computer (Supplementary Figure 1C). An average of 150 kdyne contusion force was obtained for each sample. These results indicated that the C7 spinal cord hemi-contusion model was successful. Based on myelin staining, the myelin sheath area in the SCI_KD group was significantly larger than that in the spinal cord injury with standard diet (SCI_SD) group (Figure 1B–1C), indicating that SCI rats exhibited protection or promotion of myelin sheath growth at the injured site after feeding with a KD.

### Differentially expressed mRNAs

To further understand the effects and mechanism of the KD on SCI, RNA-seq was performed on each group with 5 biological replicate samples, in which 5 samples were degraded and eliminated. To explore transcription factor expression in Sprague-Dawley rats treated with the KD under spinal cord injury, we established 15 RNA-seq cDNA libraries from the rats divided into 4 groups (SCI_KD, SCI_SD, Sham_KD, and Sham_SD). A summary of the sequence assembly after Illumina sequencing is presented in Supplementary Table 1. The average of raw reads was 23295214.7, the Q30 basic mass fraction of the sample was 100%, and the error rate (%) was 0, meeting the requirements for subsequent analysis. Using a comparison of the transcriptome and reference genome, greater than 82% of reads were uniquely mapped; these results met the subsequent analysis requirements (Supplementary Table 2). In short, the sequencing data were suitable for subsequent data analysis.

**Figure 2:**
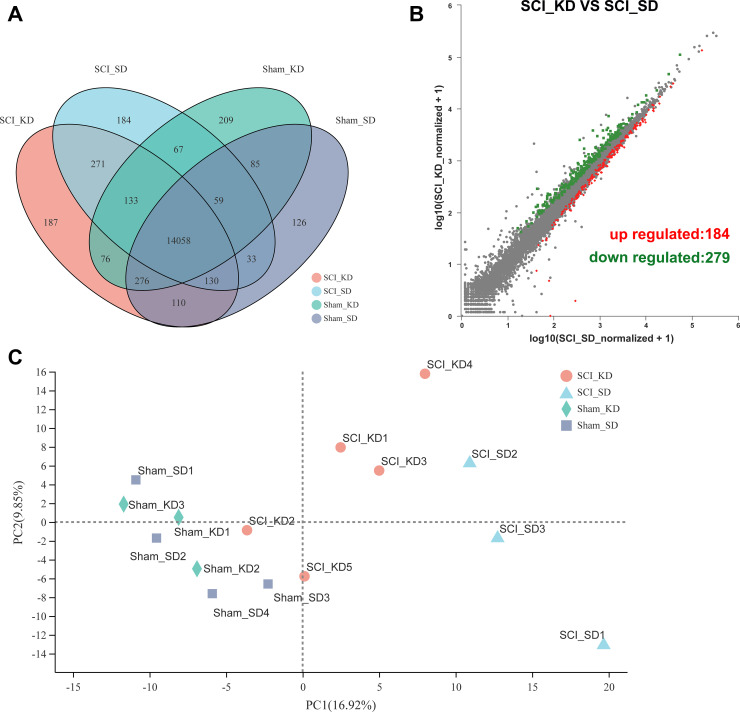
**Changes in mRNA expression profiles between groups.** (**A**) The mRNA expression of the four groups in the entire transcriptome. (**B**) Volcano plots showing the up- and downregulated mRNA transcripts in the SCI_KD vs. the SCI_SD group. (**C**) Principal component analysis (PCA) score plot with the two principal components (PC1 = 16.92%; PC2 = 9.85%).

A total of 32,883 genes were detected by RNA-seq, and Venn analysis between samples showed the number of expressed genes in each group (Figure 2A). We first used an unsupervised classification method-principal component analysis (PCA) to characterize the differences among the gene expression profiles of the 15 samples. Figure 2C shows that the biologically replicated samples could be divided into the following 4 groups: Sham_SD1-3, Sham_KD1-4, SCI_SD1-3 and SCI_KD1-5. A PCA model showed that the maximum variation in PC1 could explain 16.92% of the variation, while principal component 2 explained 9.85% of the variation [11]. In Figure 2B, the scatter plot of differential genes between the SCI_KD and SCI_SD groups shows that 463 genes were differentially expressed between the two groups, among which 184 DEGs were upregulated, and 279 DEGs were downregulated. The top 50 upregulated and downregulated genes are listed in Supplementary Tables 3 and 4, respectively.

### Reprogramming steroid biosynthesis and metabolism in SCI rats on KD

**Figure 3:**
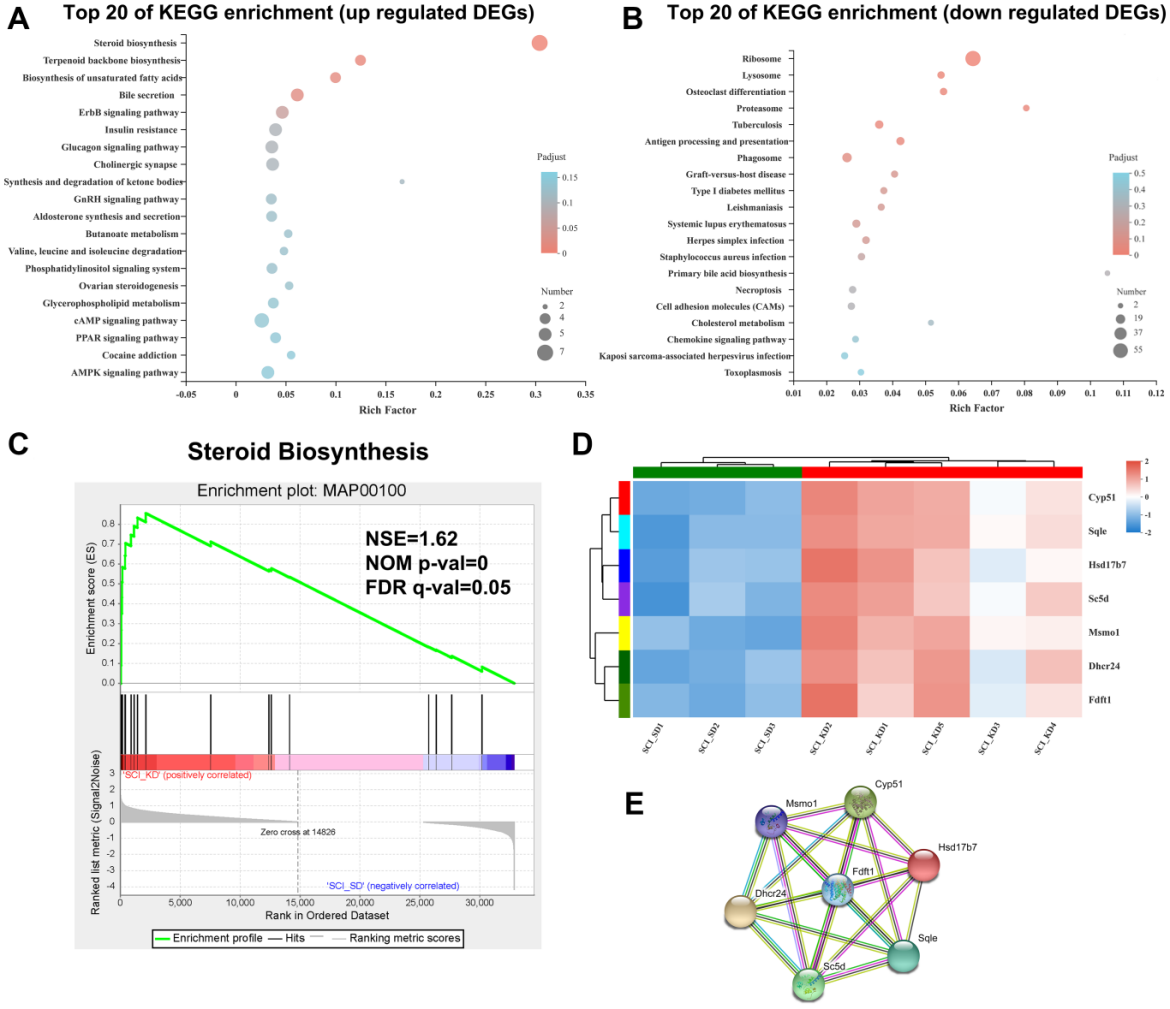
**Enrichment analysis of DEGs in the SCI_KD vs. the SCI_SD group.** (**A**) KEGG enrichment bubble diagram of the upregulated DEGs from the SCI_KD and SCI_SD groups. (**B**) KEGG enrichment bubble diagram of the downregulated DEGs from the SCI_KD and SCI_SD groups. (**C**) The GSEA analysis diagram of steroid biosynthesis between SCI_KD and SCI_SD. (**D**) Cluster analysis of KEGG-enriched DEGs. (**E**) Protein-protein interaction of DEGs between the SCI_KD and SCI_SD groups.

Compared with the SCI_SD group, the KD group exhibited upregulated DEGs in many enrichment pathways, such as the steroid synthesis pathway in lipid metabolism (path ID: map00100, *q*-value = 5.82043E-08), biosynthesis of unsaturated fatty acids, terpenoid backbone biosynthesis, etc. A bubble chart of the DEGs in the top 20 most enriched pathways is provided (Figure 3A). The downregulated DEGs from the KD diet group were significantly enriched in ribosomes, lysosomes, osteoclast differentiation, proteasome, tuberculosis, and antigen processing and display. Figure 3B shows the KEGG enrichment distribution of the DEGs.

To further understand the biological functions of the SCI_SD and SCI_KD DEGs, we performed gene set enrichment analysis (GSEA) of the sample sets based on the C2 MSigDB database, which includes the Broad Institute recombined gene sets. The selected gene set was tested, and it was found that SCI_KD was significantly negatively correlated with inflammatory cytokines and their receptors (the NES values were ˗1.44 and ˗1.46, respectively; Supplementary Figure 2), suggesting that a KD reduces the lethality of SCI, which echoes the results of our previous studies. Similarly, based on the KEGG functional annotation, we found that SCI_KD was significantly positively correlated with the steroid synthesis pathway (the NES is 1.62; Figure 3C) by GSEA analysis, which further shows that the KD is involved in the metabolic reprogramming of steroids in SCI rats, which is a positive effect of the KD. An important mechanism of SCI function may be related to reducing metabolic responses. iPath3.0 was used to visually analyse the enriched steroid metabolism-related genes, as shown in Supplementary Figure 3, which shows that the steroid metabolism DEGs in the biological system transmit information.

### Cluster analysis and PPI analysis of DEGs in the steroid synthesis pathway

Figure 3D shows that the *Sqle* (squalene epoxidase), *Sc5d* (sterol-C5-desaturase), *Cyp51* (cytochrome P450, family 51), *Dhcr24* (24-dehydrocholesterol reductase), *Msmo1* (methylsterol monooxygenase 1), *Hsd17b7* (hydroxysteroid (17-β) dehydrogenase 7), and *Fdft1* (farnesyl diphosphate farnesyltransferase 1) genes were obviously highly expressed in the SCI_KD group. DEGs corresponded to interaction relationships for network integration and allowed us to identify key pathways in the interaction network according to indicators such as the connectivity between genes (Figure 3E).

### WGCNA and hub module identification and analysis

**Figure 4:**
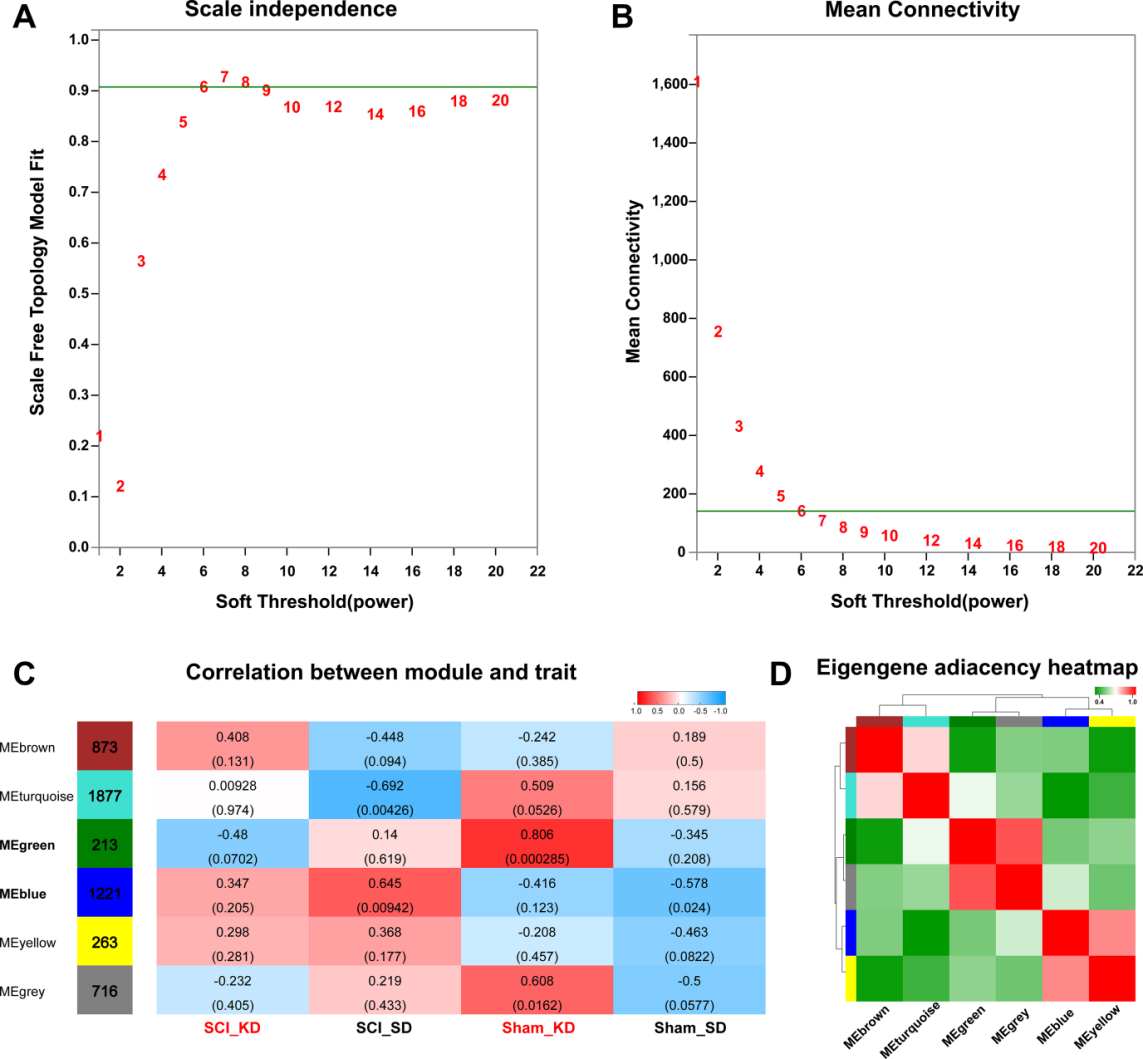
**Data pre-processing and module identification for WGCNA analysis.** (**A–B**) Analysis of the network topology with different soft threshold powers. The left panel shows the influence of soft threshold power on the scale-free topology fitting index. The right panel shows the influence of soft threshold power on the average connectivity. (**C**) Analysis of the correlation between the module and the diet phenotype. Red indicates that the module has a higher correlation with the phenotype, while blue means the module has a lower correlation with the phenotype. (**D**) WGCNA module correlation; the colour indicates the correlation between modules.

According to the KEGG enrichment analysis of differential gene expression and the GSEA, the mean values showed that SCI rats in the KD group exhibited significantly increased steroid anabolism, which indicates that the KD may be protective by mediating steroid metabolism reprogramming. By changing certain physiological functions or pathways, we could analyse the SCI_KD, SCI_SD, Sham_KD, and Sham_SD results in a gene network (WGCNA) and fully understand the roles of a KD and standard diet (SD) on genetic changes in Sprague-Dawley rats. Genes that were commonly expressed in the four groups were placed in the same gene network through the WGCNA algorithm. A total of 32,883 mRNAs were screened by WGCNA, and a total of 5,163 mRNAs were used for subsequent analysis after pre-processing and filtering, such as excluding deletions and outliers. Figure 4A and 4B show the scale-free fitness curve and the average connectivity curve, respectively. The scale-free fitness curve is in a smooth position, and the power exponent is a weighted β value; thus, the soft threshold value is set to 6, and the scale-free topological index is 0.9. Therefore, the network conforms to the power law distribution and is close to the actual biological network state. The resulting gene tree diagram and the colours in the corresponding modules are shown in Supplementary Figure 4A–4B, with the number of mRNAs for each module shown in Supplementary Table 5.

After the data were pre-processed, the genes were classified, and genes with similar expression patterns were divided into categories called modules. We performed module clustering and module correlation analysis for the obtained modules, as shown in Figure 4D. Interaction relationships were analysed in the six different modules, and a network heat map of the correlation between the modules and the phenotype was drawn (Figure 4C). The results show that the modules are independent of one another, which indicates the relative independence of gene expression in different modules. Here, we mainly analysed the correlation modules between the KD- and SD-fed rats. Among them, the MEgreen module is highly related to a KD, and the MEblue module is highly related to a SD. We then calculated the correlation coefficient between each gene in the module and specific phenotype data to obtain the gene significance (GS) value; this value was combined with the MM value of each gene in the module (i.e., kME, eigengene-based connectivity) to obtain the MM-GS scatter plot. The stronger the correlation, the closer the relationship between the module and the phenotype; moreover, correlations with a lower p-value are more reliable. This analysis shows that MEgreen is an important module in the KD, while MEblue is an important module in the SD (Supplementary Figure 4C–4F).

### Functional annotation analysis of hub modular genes

**Figure 5:**
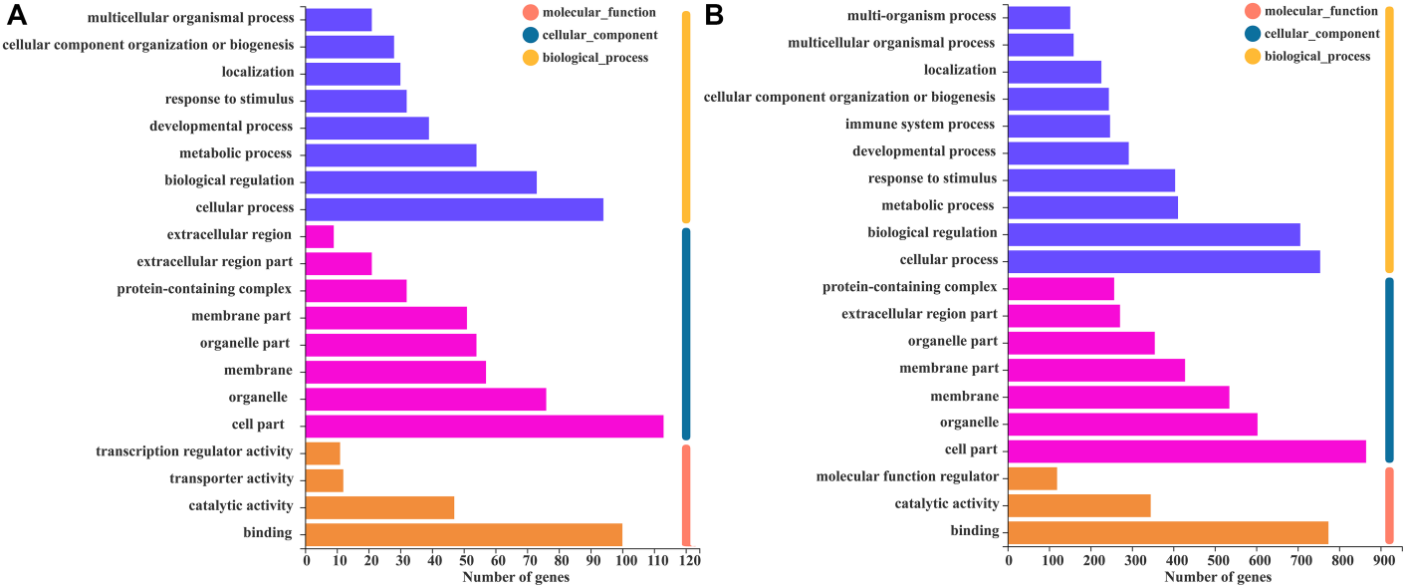
**GO function annotation analysis of WGCNA module genes.** (**A**) The GO function annotation analysis histogram of the MEgreen module. (**B**) The GO function annotation analysis histogram of the MEblue module, which includes biological processes, cell components, and molecular functions.

To explore the biological functions of the KD and SD modules (green and blue, respectively) and compare the differences in their biological functions, we performed GO and KEGG enrichment analysis with annotation and visualization. GO function annotation analysis classifies DEGs using different perspectives, such as the biological processes involved, the components that constitute cells, and the molecular functions achieved. Figure 5A shows the GO annotation functions of the MEgreen module, and Figure 5B shows the GO annotation functions of the MEblue module. Different from other groups, there was a significant positive correlation between SCI rats that received a SD and the MEblue module. GO functional analysis of immune system processes is expressed as biological processes, which indicated that the immune system significantly affected SCI rats, and immune system processes included all processes of inflammation-related pathways and congenital and acquired immune expression changes.

Similarly, KEGG annotation analysis was also performed on the green and blue modules. The results showed that the KEGG annotation molecule in the MEblue module significantly identified immune system, immune system and infectious diseases: viruses and other immune disease pathways among the subtypes. This module mainly focused on rats with SCI that received a SD, which is consistent with the GO function analysis results. Compared with the MEblue module, in terms of expression ratio, immune system, immune system and infectious diseases with the sub-categories of viruses and other immune disease pathways were significantly downregulated, and the module is related to the KD (Supplementary Figure 5).

### Multi-gene set KEGG enrichment analysis and gene interaction network analysis

**Figure 6:**
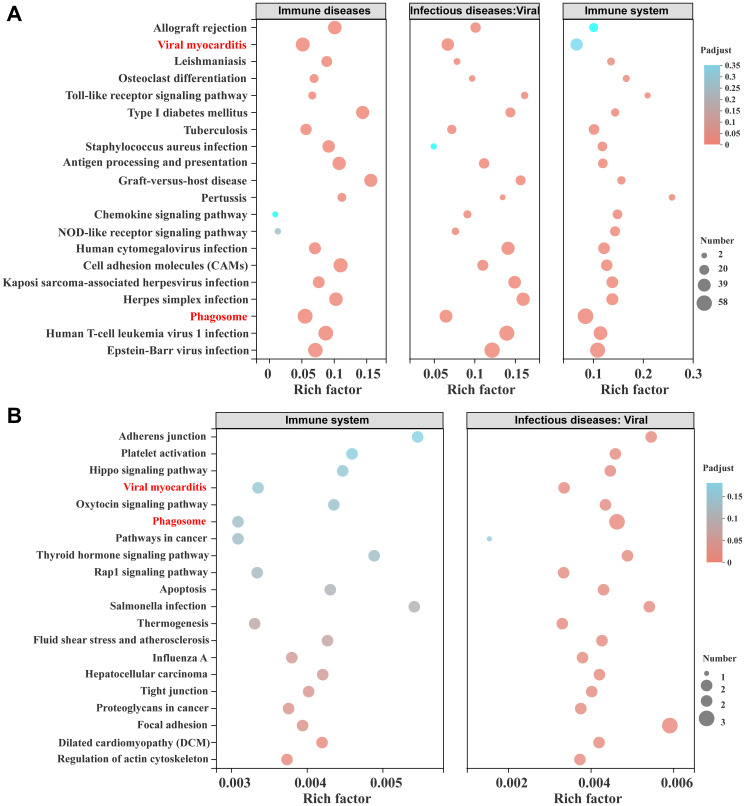
**Multi-gene set KEGG enrichment analysis and modular gene visualization.** (**A**) The bubble diagram of the intersection of the KEGG pathway in the MEblue module, including the immune system and infectious diseases: viral and immune diseases gene sets. (**B**) The bubble diagram of the intersection of the KEGG pathway in the MEgreen module, including the immune system and infectious diseases: viral gene sets.

The immune system and infectious diseases: viral and immune disease pathways were used as pathways of interest for multi-gene KEGG enrichment analysis. Figure 6A shows the KEGG enrichment results of the MEgreen module, and Figure 6B shows the KEGG enrichment results of the MEblue module. The MEgreen module is primarily a module closely related to the KD rats, while the MEblue module is mainly a module closely linked to the SD rats, especially the SD injury rats. The results of the KEGG enrichment signal pathways and intersection of the top 20 pathways for the two modules show that only the viral myocarditis and phagosome pathways are present in both modules. The MEgreen module mainly activates innate immune-related pathways, such as the Toll-like receptor signalling pathway and the NOD-like receptor signalling pathway. These results indicate that MEgreen and MEblue act on different immune pathways. The WGCNA and functional analysis showed that rats fed a KD showed a reduction in immune system pathway processes, including immune diseases, infectious diseases and other immune microenvironments.

## DISCUSSION

The KD has been widely used in the treatment of a variety of neurological and metabolism-related diseases, especially epilepsy [22–23]. In recent years, some studies, including previous studies performed by our team, have shown that the KD has a protective effect after SCI by increasing spontaneous movement and fine manipulation in SCI animal models [17, 24–25]. However, the mechanism of action of the treatment is still unclear. In this study, myelin expression and transcriptome level changes between different groups were detected in rats under different dietary feeding methods. This study is the first to use RNA-seq to understand and identify DEGs in SCI rats fed a KD and then examine which biological signalling pathways can explain the protective effects of the KD diet on SCI rats.

We used a variety of analytical methods to mine and interpret entire transcriptome RNA-seq data, including DEG and functional enrichment analysis between groups, GSEA between groups, iPath metabolic pathway analysis, gene cluster analysis, WGCNA and module analysis, module function analysis and gene interaction network analysis. From single-gene expression analysis to changes in the overall gene network, a comprehensive analysis of the changes in gene expression patterns under a KD can provide a deeper understanding of the changes in gene transcription and signal pathways induced by a KD for the treatment of SCI.

From a local perspective, we used KEGG pathway enrichment of upregulated DEGs in the SCI_KD and SCI_SD groups and GSEA analysis of these groups to reach a consistent conclusion that KD significantly increased the steroid anabolic pathway in rats with SCI. Through cluster analysis, PPI analysis and the iPath metabolic pathway visualization, we concluded that *Sqle, Sc5d, Cyp51, Dhcr24, Msmo1, Hsd17b7,* and *Fdft1* expression changed significantly in the pathway. Steroid biosynthesis appears to play a pivotal role in SCI.

The KD is a high-fat, low-carbohydrate and moderate-protein diet. Its purpose is to reduce carbohydrate intake and replace it with lipids, while ensuring a sufficient protein supply [26]. This programme was proposed by Wilder in 1921 for the treatment of epilepsy. It was later used for a variety of neurological diseases, such as Alzheimer's disease, Parkinson's disease, etc. [9, 27]. Although it is known that a KD promotes high ketone body levels, high fat, low carbohydrates, low calories and other "immediacy" properties [28–29], this study suggests that the KD may mediate steroid metabolism reprogramming to treat SCI in rats. As an alternative method to transiently raise systemic ketone body levels, most individuals practice the KD, this dietary approach can (1) decrease hepatic de novo lipogenesis; (2) alleviate low-grade chronic inflammation; (3) abate hyperglycemia; (4) elevate systemic β-hydroxybutryate levels, which reflects an increase in β-oxidation; and (5) promote the abundance of folate-producing Streptococcus in the gut, which further up-regulates folate-dependent one-carbon (-CH3) metabolism [30].

This study is the first to reach this conclusion. Steroids include sterols (such as cholesterol, lanosterol, sitosterol, stigmasterol, and ergosterol), bile acids and bile alcohols, steroid hormones (such as adrenal cortex hormones, androgens, and oestrogen), insect ecdysone, cardiac glycosides, saponins, and toad poison [31–33]. Eating a high-fat/low-carbohydrate KD can cause the liver to produce ketones. In the brain, ketone bodies (such as β-hydroxybutyrate) contribute to sterol synthesis [34], which is essential for the growth of myelin membranes [35]. Studies have shown that diabetic patients can significantly increase HDL cholesterol (HDLc) and lower blood pressure through a 90-day KD, while LDL cholesterol shows no significant difference [36]. However, long-term KD consumption increases the risk of type 2 diabetes and metabolic syndrome. This study explored the relevant mechanism of short-term KD treatment in SCI rats [37]. Additionally, studies have found that HDLc showed small increases after ketogenic phases, but there was no significant change over 12 months [38].

This study also found that myelin areas were significantly larger in SCI rats after being fed a KD than in SCI rats in the SD group. A recent study by Stumpf found that a KD can improve axon defects and promote myelination in Pelizaeus-Merzbacher disease. A high-fat/low-carbohydrate KD can restore the integrity of oligodendrocytes and increase CNS myelin. Lipids (such as ketone bodies) easily enter the CNS without destroying the blood-brain barrier or blood-spinal cord barrier [39]. Ketone bodies produced by a SD are not able to enter the CNS to enhance the myelin membrane. These conclusions indicate that a KD mediates steroid metabolism reprogramming after SCI, facilitates myelin membrane synthesis, promotes myelin sheath regeneration, plays a unique role in neuroprotection, and does not destroy the blood spinal cord barrier function. It is well known that the blood spinal cord barrier is an important barrier for the spinal cord to maintain neuroimmune balance.

This study combined 4 groups of rat genes into the same gene network through WGCNA to analyse the correlation between gene modules and phenotype. Through module identification and analysis as well as GO function and KEGG pathway analyses, we showed that rats fed a KD displayed significantly reduced immune-related pathways, including immune diseases and infectious diseases. In addition, examining the data from the SCI_KD and SCI_SD groups and existing MSigDB analysed by GSEA, we concluded that SCI_KD is significantly negatively correlated with inflammatory cytokines and their receptors. These results indicate that KD treatment can improve the body's immunity and reduce infection and inflammation.

In SCI secondary injury, inflammation is an important and main pathophysiological process. After SCI, ischaemia-reperfusion injury, endothelial cell injury and homeostasis disorders jointly initiate the inflammatory cascade reaction, and spinal cord innate immune cells (microglia and astrocytes) [40] and infiltrating white blood cells (neutrophils and macrophages) are activated [40–41]. The activated inflammatory cells release a large number of inflammatory factors to further expand the damage and increase the time and space of the damage response. Our research has shown that KD reduces or inhibits neutrophils in the plasma of SCI rats and inhibits the classic NF-κB pro-inflammatory signalling pathway at the injury site, which corresponds to the results of this study [17]. Steroid synthesis is also closely related to SCI neuroinflammation, and steroid synthesis hormones [42], oestrogen [43–44], saponin [45], tauroursodeoxycholic acid [46] and other steroid components in SCI all have anti-inflammatory properties. Figure 6A–6B show the KEGG signalling pathway at the intersection of the immune microenvironment of a SD and a KD, respectively. The SD mainly activates innate immune-related pathways, such as the Toll-like receptor signalling pathway and NOD-like receptor signalling pathway. This study cannot directly prove a correlation between KD-mediated reprogramming of steroid metabolism and neuroimmunity, which is the limitation of the study. The significantly expressed genes in the steroid synthesis pathway need to be further examined in future studies to determine if they are related to SCI efficacy.

## CONCLUSIONS

This study is the first to demonstrate that the KD significantly promotes the reprogramming of steroid metabolism in the treatment of SCI at the transcriptional level. These changes are beneficial for myelin sheath growth and promote nerve function. Moreover, a comprehensive analysis of the KD through WGCNA indicates the improvement of the SCI immune microenvironment and reduction in inflammation. Therefore, we believe that the KD may improve the immune microenvironment and myelin growth in rats with SCI through the reprogramming of steroid metabolism.

## METHODS

### Animals and diets

All animal experiments conformed to the Guide for the Care and Use of Laboratory Animals (NIH Publications No. 8023, revised 1978). All procedures and euthanasia performed in this study were approved by the Ethics Committee for Animal Experiments of the Department of Laboratory Animal Science, Peking University Health Science Center (No. LA201456, 24 February 2014).

We randomly divided 32 8-week-old male Sprague-Dawley rats (300–360 g) into the following four groups (*n* = 8 in each group): the control group was undamaged and divided into the SD group (Sham_SD) and the KD group (Sham_KD), while the SCI rats received either the SD (SCI_SD group) or KD (SCI_KD group); 20 rats were used for RNA-seq, and 12 rats were used for histopathological examination of fixed specimens. All rats were reared at 21°C with a light/dark cycle of 12/12 hours, and food and water were provided freely. Rats in the SCI_KD group were fed a KD (70 g/kg body weight/day), and the ratio of fat to carbohydrate and protein was 4:1 (fat: 50.5 g/100 g KD; carbohydrate: 23.6 g/100; four hours after SCI, protein content: 5.5 g/100 g KD; dietary fibre: 16.1 g/100 g KD (Shenzhen Zeneca Inc., Shenzhen, China)). The rats in the Sham_SD group and SCI _SD group were fed normal carbohydrates. All groups had free-access water supply.

### C7 spinal cord semi-contusion

A C7 hemi-contusion was performed in the two SCI groups as described previously for a different site of contusion [20]. Briefly, rats were anaesthetized with sodium pentobarbital after a unilateral (left or right side, chosen randomly) C7 laminectomy, and the C6-T1 vertebrae were rigidly fixed in a frame tilted at a 25.0° angle. We then used an Infinite Horizon impactor to deliver a set contusion force of 150 kdyne [21]. The C7 hemi-contusion is hereafter described as ‘SCI.’ Rats were fed for four weeks according to the above grouping and diet plan, and spinal cord tissue was sampled after four weeks.

### EC myelin staining

Spinal cord tissue was placed in a fully automatic tissue dehydrator for an overnight dehydration programme (xylene, alcohol isocratic dehydration), embedded in paraffin, and cut it into 5-μm continuous sections with a microtome after cooling overnight at 40°C. The tissues were soaked in xylene I and II for 30 minutes each, after which they were soaked in 100%, 100%, 95%, 95%, and 80% alcohol for 5 minutes each and then rinsed twice with double-distilled water. The tissues were then stained with eriochrome cyanine (EC) for 15–20 minutes before undergoing ferric chloride (5.6%) differentiation for a specific time under a microscope. We made sure not to over-differentiate, as the staining would be too light. Afterwards, the samples were dehydrated and mounted with neutral gum.

### RNA isolation, RNA-seq library construction and sequencing

We randomly divided 20 Sprague-Dawley rats into 4 groups: SCI_KD; SCI_SD; Sham_KD; and Sham_SD. Total RNA was extracted from a 1.6-cm spinal cord segment at the centre of the impact site with the TRIzol reagent (Invitrogen, Thermo Fisher Scientific Inc., MA, USA) and subjected to RNA-seq analysis to assess gene expression (*n* = 5). Genomic DNA was removed using DNase I. Agarose gels (1%) were used to monitor RNA degradation and contamination. A NanoPhotometer^®^ spectrophotometer (IMPLEN, CA, USA) was used to check RNA purity. A Qubit^®^ RNA Assay Kit was used to measure the RNA concentration on a Qubit^®^ 2.0 Fluorometer (Life Technologies, CA, USA). An RNA Nano 6000 Assay Kit was used to assess the RNA integrity on a Bioanalyser 2100 system (Agilent Technologies, CA, USA). RNA sequencing of 5 biological replicate samples was performed for each group, and 5 samples were degraded and eliminated. Therefore, 15 RNA-seq cDNA libraries were established.

1) We utilized two methods to treat the total RNA. Oligo(dT) magnetic beads were used to select mRNAs with polyA tails. rRNAs were hybridized with DNA probes and then digested to generate a DNA/RNA hybrid strand, followed by DNase I digestion to remove DNA probes. The target RNAs were then obtained by purification. 2) The target RNA was fragmented and then reverse transcribed into double-stranded cDNA (dscDNA) with N6 random primers. 3) The dscDNA was end-repaired with phosphates at the 5′ end and sticky ′A′s at the 3' end, after which the sequence was ligated to an adaptor with sticky 'T's at the 3' end of the dscDNA. 4) Two specific primers were used to amplify the ligation product. 5) The PCR products were denatured with heat, and the single strand DNA was cyclized with a splint oligo and DNA ligase. 6) Finally, the prepared library was sequenced with the Illumina HiSeq xten (2 × 150-bp read length, Illumina, San Diego, CA, USA). The sequencing programmes were performed by BGI Company (China, Shenzhen).

### Quality control and expression level

The raw paired-end reads were trimmed and subjected to quality control by SeqPrep (https://github.com/jstjohn/SeqPrep) and Sickle (https://github.com/najoshi/sickle) with default parameters. The paired-end clean reads were aligned to the Rattus_norvegicus reference genome (Rnor_6.0) using the default parameters in TopHat (http://tophat.cbcb.umd.edu/, version 2.1.1) [47]. The reference genome and gene model annotation files were directly downloaded from genome website (http://www.ensembl.org/Rattus_norvegicus/Info/Index). RSEM (http://deweylab.biostat.wisc.Edu/rsem/) [48] was used to quantify the gene abundances. To identify DEGs between two different samples, the expression levels of each transcript were calculated based on the number of transcript reads per million reads (TPM). The reads were then normalized using DESeq2 version 1.24.0 (http://bioconductor.org/packages/stats/bioc/DESeq2/) [49], and the statistical significance of the DEGs was assessed. The following default parameters and criteria were used here: Benjamini & Hochberg (BH) p-adjust < 0.05 and |log2FC|≥1 after multiple comparisons. In the initial data exploration, we performed principal component analysis (PCA) to directly calculate the coefficient of variation between the groups in R ‘prcomp’.

### Gene function annotation analysis

The genes and transcripts are aligned to six databases (NR, Swiss-Prot, Pfam, EggNOG, GO, and KEGG) to obtain comprehensive annotation information. DIAMOND software (https://github.com/bbuchfink/diamond) [50] was used to sequence the genes and transcripts with the NR (ftp://ftp.ncbi.nlm.nih.gov/blast/db/), Swiss-Prot (ftp://ftp.uniprot.org/pub/databases), and EggNOG (http://eggnogdb.embl.de/#/app/home) databases [51]. Sequence alignment was performed with the BLAST2GO and GO database, followed by alignment with the Pfam database using HMMER software [52]. GO (http://www.geneontology.org/) is a database established by the Gene Ontology Federation. Its purpose is to standardize the biological terminology of genes and gene products in different databases and to determine gene and protein functions. Using the GO database, the genes in the selected gene set can be classified according to the biological process (BP) they participate in, the cell component (CC), and the molecular function (MF) they achieve. The results of KEGG Orthology (Kyoto Encyclopaedia of Genes and Genomes, http://www.genome.jp/kegg/) were obtained using KOBAS2.1. KEGG is a knowledge base that systematically analyses gene functions and links genomic information and functional information. Using the KEGG database, the genes in the gene set can be classified according to the pathways involved or the functions they perform [53].

### KEGG pathway enrichment analysis

The KEGG PATHWAY enrichment analysis was performed on the gene set using an R script in KOBAS (http://kobas.cbi.pku.edu.cn/home.do) [53]. The BH method was used to calculate the corrected *p* value. The threshold of the corrected *p* value (corrected *P*-value) was 0.05. The KEGG pathway that met the conditions showed that the pathway was significantly enriched for the gene set.

### iPath metabolic pathway analysis

iPath3.0 (http://pathways.embl.de) was used to visually analyse the metabolic pathways and facilitate observation of the metabolic pathway information of DEGs in the entire biological system. Nodes represent various biochemical molecules, and lines represent biochemical reactions. The overall metabolism of the biological system includes 146 metabolism-related KEGG pathways, 22 regulatory-related KEGG pathways, and 58 KEGG pathways involved in the biosynthesis of secondary metabolites. The nodes in the figure represent different compounds, and the boundaries represent different enzymatic reactions. iPath3.0 can outline the biosynthesis and important regulatory pathways of secondary metabolites and easily identify complex metabolic pathways [54].

### Gene set enrichment analysis (GSEA)

GSEA is performed on a predefined set of genes, usually from functional annotations or the results of previous experiments (included in MSigDB, version 6.2, http://software.broadinstitute.org/gsea/downloads.jsp), and organizes the genes according to two sample types. The GSEA sorts the degree of differential expression and then checks whether the gene set clusters at the top or bottom of the ranking list. The first round of enrichment is based on C2, which includes curated gene sets (from online pathway databases, publications in PubMed, and knowledge of domain experts). The second enrichment is based on the results of KEGG functional annotation in this study. For the analysis results, we generally considered that gene sets with the following characteristics were meaningful: |NES|>1, NOM *p*-val<0.05, and FDR *q*-val<0.25.

### Protein-protein interaction (PPI) analysis

We used an online search tool (STRING database, version 10.5; http://string-db.org/) to search for interacting genes to construct a protein-protein interaction (PPI) network based on the extensive functions of the genome-wide data [55].

### Weighted gene co-expression network analysis (WGCNA)

A co-expression network was constructed using the WGCNA (version 1.47) package in R [21]. WGCNA is an undirected, weighted network. "Unweighted network" means that the correlation between genes can only be 0 or 1. 0 means that the two genes are not related, while 1 means they are related. A "weighted network" means not only that genes are related or not but also that their correlation value is recorded. The value is the weight (correlation) of the connection between genes. The soft threshold (β power) in the co-expression network is the expression of each gene at a specific time or space and is regarded as a node. To obtain the correlation between genes, it is necessary to calculate the correlation coefficient (Pearson coefficient) any two genes; this allows us to know whether the expression profiles of two genes are similar. In this study, the β power = 6.

1) The module refers to the grouping of genes with similar expression patterns. Genes of a specific type below to a module.

2) An eigengene is defined as the first principal component of a given module. It is the most representative expression pattern of the module. It is an indicator that approximately represents the expression status of all genes in a module but is not a real gene.

3) A hub module refers to a module that is highly related to phenotypic data or closely related to research.

4) Gene significance (GS), or a measure of gene importance, is the correlation between gene expression patterns and the characteristics of a sample. Generally, the larger the correlation value, the more important the gene, which can be evaluated as the log conversion of the *p*-value. The correlation index between a gene and a specific pathway can also be the correlation between a gene and a specific phenotypic feature.

5) Module membership (MM), also known as the eigengene-based connectivity (kME), is the correlation between the expression pattern of a gene and a characteristic gene in the module. The larger the value, the greater the possibility that the gene belongs to the module.

Genes with the highest MM and the highest GS are genes with high significance (hub genes). All these analyses were performed using commands implemented in the WGCNA software package with the following parameters: minModuleSize = 30, minKMEtoStay = 0.3, and mergeCutHeight = 0.2.

### Modular gene expression correlation analysis

After a gene of interest is obtained based on the correlation of gene expression, the correlation coefficient between genes is determined using the Spearman correlation algorithm. The correlation coefficient threshold is 0.5, the significance level is the *p* value after BH correction (corrected *p*-value) ≤0.05, and these values were drawn into a visual network diagram. In the figure, the nodes represent genes, and the connections between nodes represent the correlation between gene expression. The larger the node is, the greater the expression correlation between the genes.

### Multi-gene set KEGG pathway enrichment analysis

After performing KEGG analysis on the different gene sets, we utilized the intersection of the selected gene sets for the display and adjusted the significance level to the corrected *p*-value ≤0.5 as the threshold to display the top 20 pathways.

### Statistical analysis

The data are expressed as the mean ± standard deviation. The statistical analysis was performed with GraphPad Prism 7.0 (GraphPad Software Inc., San Diego, CA). Student's *t*-test was used for comparisons between two groups. A *p* value < 0.05 was considered statistically significant. ^*^p < 0.05, ^**^p < 0.01, ^***^p < 0.001, and ^****^p < 0.0001.

### Availability of data and materials

RNA-seq raw data have been deposited in the NCBI Sequence Read Archive (SRA, https://submit.ncbi.nlm.nih.gov/subs/sra/SUB7836712/overview). Accession IDs for *Rattus norvegicus* BioProject = PRJNA648739; BioSample  = SAMN15646629 – SAMN15646643 (15 objects); SRA = SRR12327118 – SRR12327132 (15 objects).

## Supplementary Materials

Supplementary Figures

Supplementary Tables 1, 2, 5

Supplementary Table 3

Supplementary Table 4
