# Supplementary material for: Ketogenic diet-mediated steroid metabolism reprogramming improves the immune microenvironment and myelin growth in spinal cord injury rats according to gene and co-expression network analyses
Source: Aging (Albany NY). 2021 May 6;13(9):12973–95. doi: 10.18632/aging.202969 (PMC8148504; doi:10.18632/aging.202969)
Supplement: Supplementary Figures [file aging-13-202969-s001.pdf]

SUPPLEMENTARY FIGURES

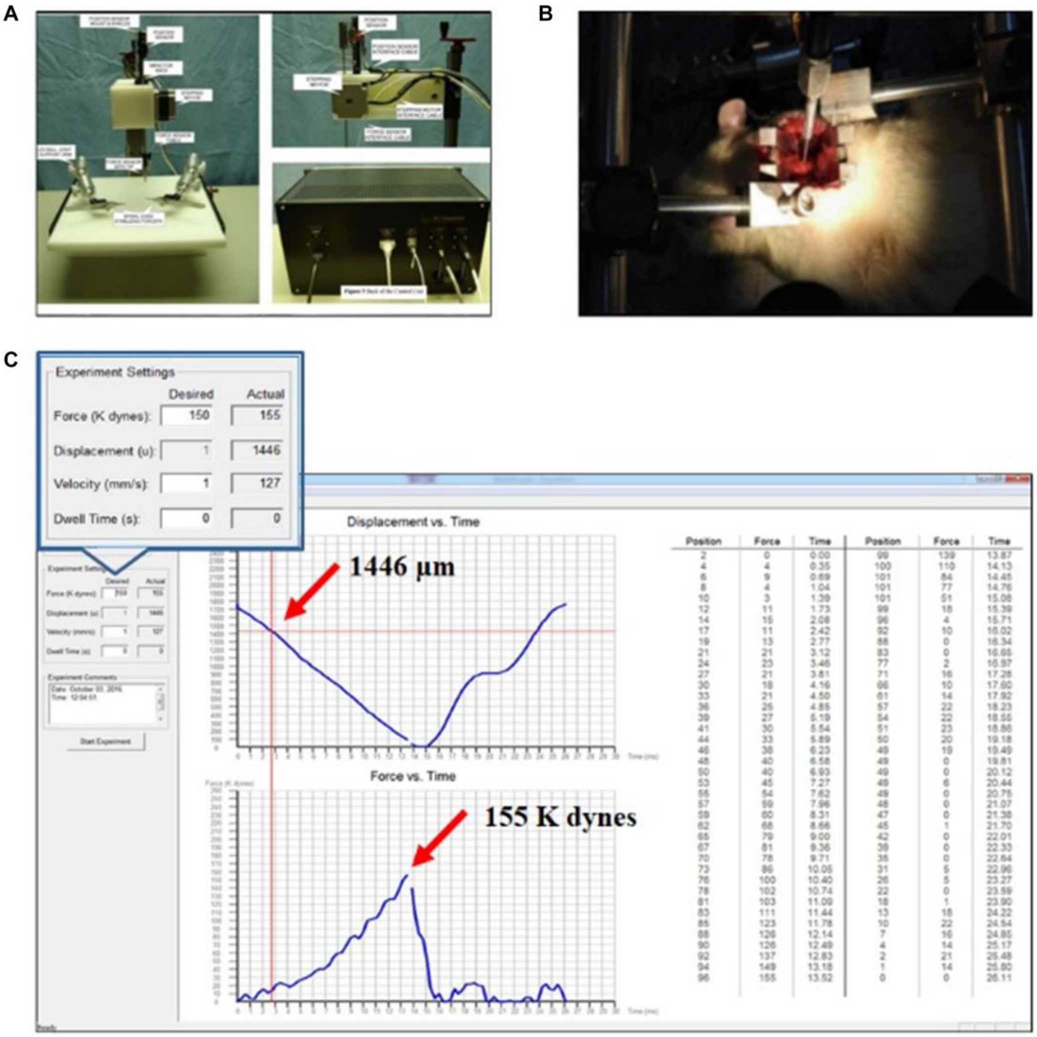

Supplementary Figure 1. Verification of C7 spinal cord hemi-contusion models. (**A**) Representative images of Infinite Horizon impactor. (**B**) Representative images of C6-T1 vertebrae fixed in a frame tilted at a 25.0° angle. (**C**) Representative images of monitoring data after striking by the Infinite Horizon impactor.

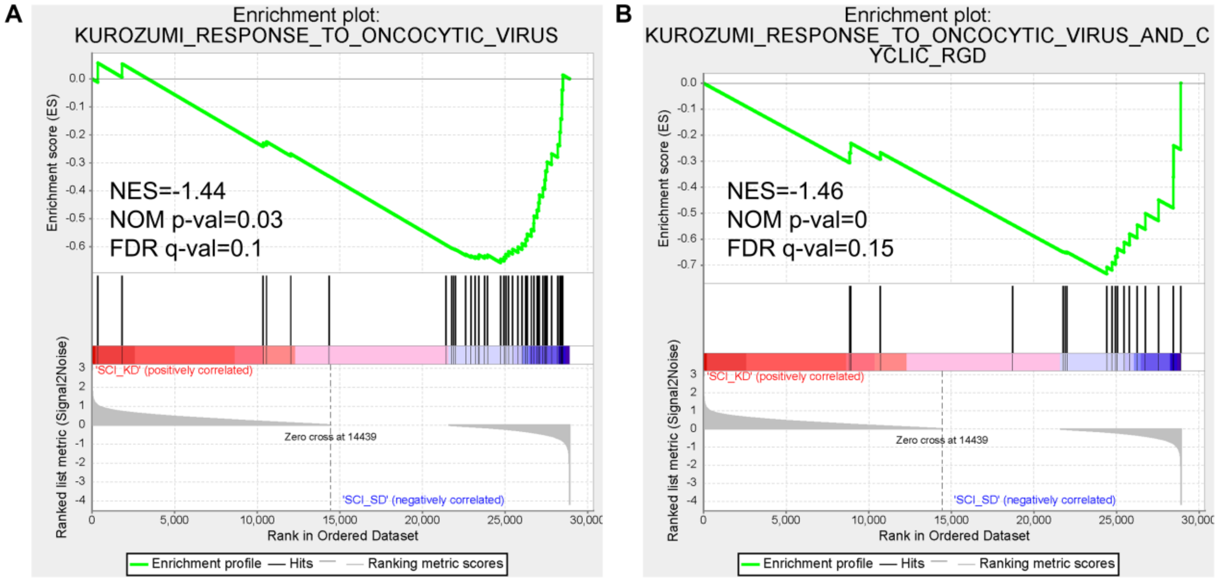

Supplementary Figure 2. GSEA showing that SCI_KD and D are significantly negatively correlated with inflammatory cytokines and their receptors, and the NES values are -1.44 (**A**) and -1.46 (**B**), respectively.

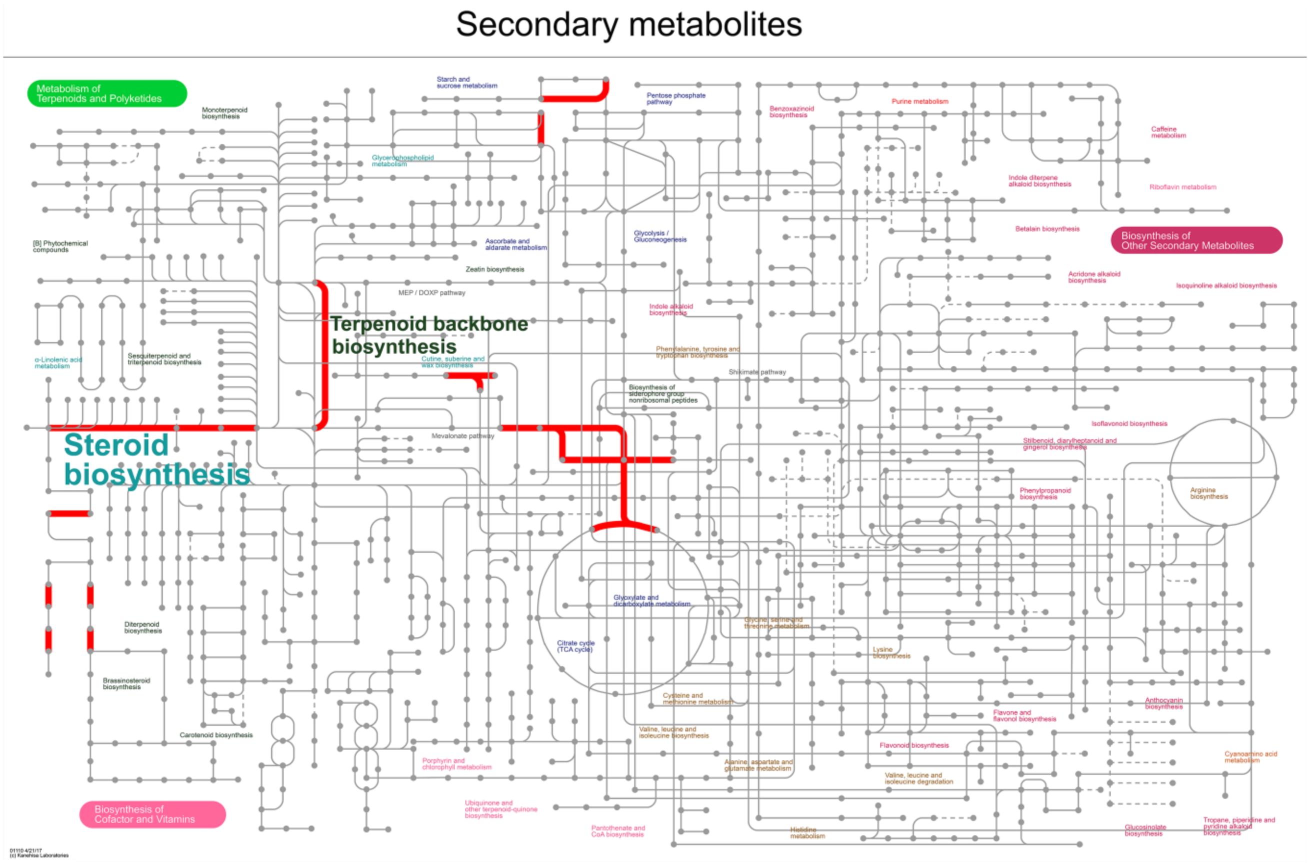

Supplementary Figure 3. Visual analysis of enriched steroid metabolism-related genes using iPath3.0.

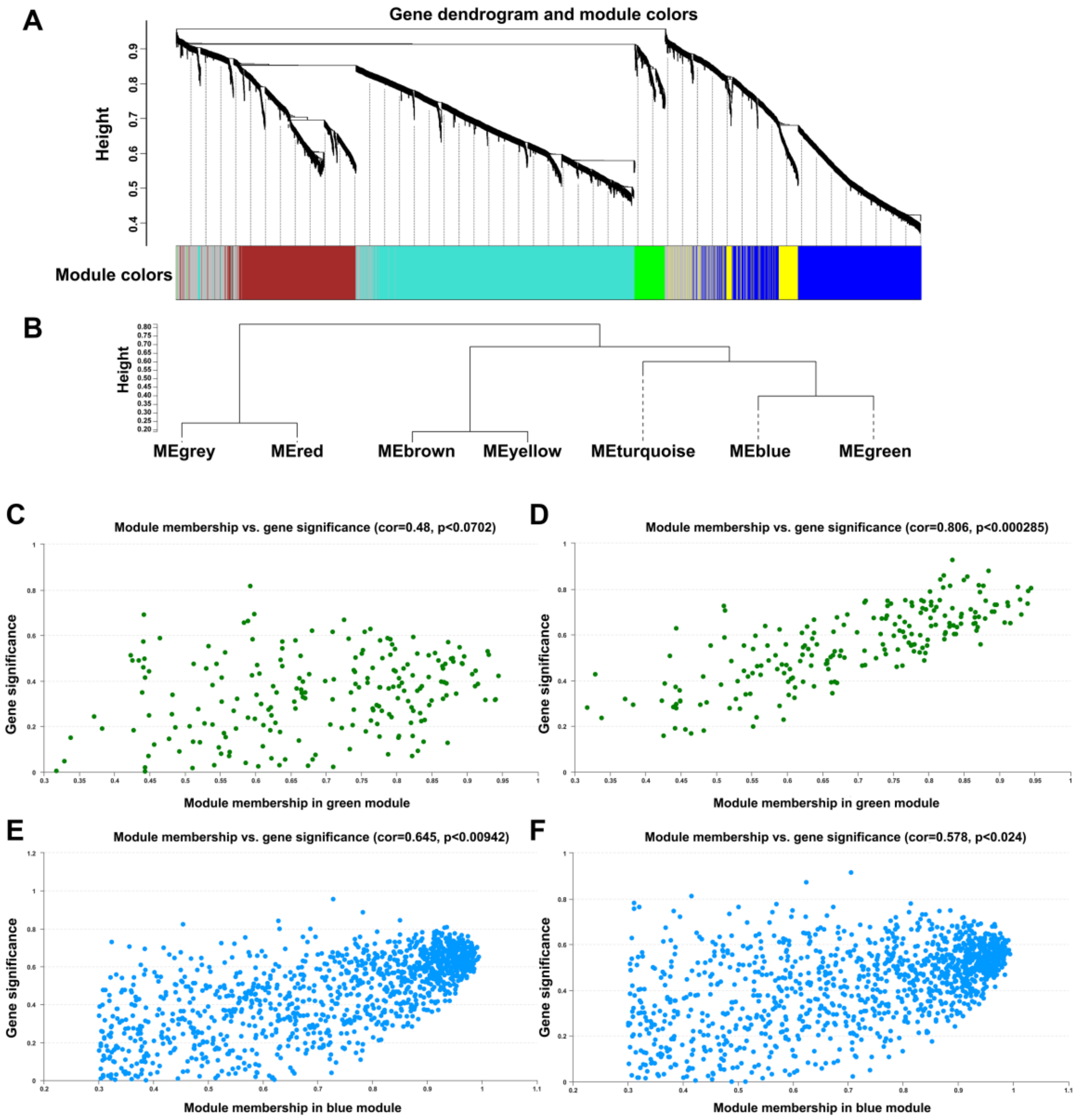

Supplementary Figure 4. Visualization of WGCNA analysis features. (**A**) Module clustering tree diagram display, in which branches represent a module. The closer two modules are branched, the closer the distance between the two modules. (**B**) Gene clustering tree (dendrogram) obtained by hierarchical clustering based on adjacent dissimilarity. (**C**–**D**) MM-GS analysis of the MEgreen module, in which C shows the SCI group, and D shows the Sham group. (**E**–**F**) MM-GS analysis of the MEblue module, in which E shows the SCI group, and F shows the Sham group.

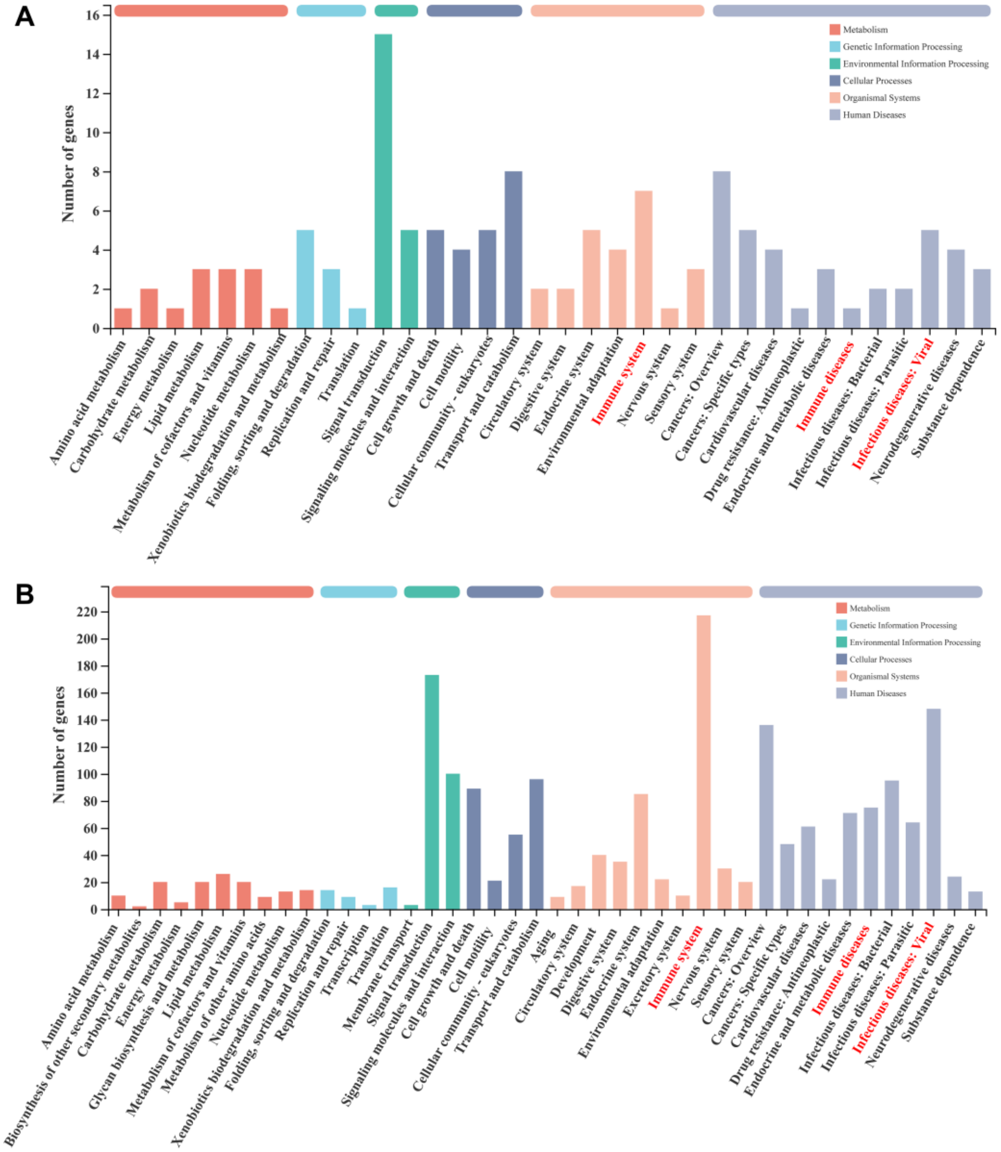

Supplementary Figure 5. Analysis of WGCNA module genes by KEGG pathway annotation. (**A**) The KEGG pathway annotation analysis histogram of the MEgreen module. (**B**) The KEGG pathway annotation analysis histogram of the MEblue module, in which the red font indicates items with significant changes in the green and blue modules.
