# Supplementary material for: Ketogenic diet-mediated steroid metabolism reprogramming improves the immune microenvironment and myelin growth in spinal cord injury rats according to gene and co-expression network analyses
Source: Aging (Albany NY). 2021 May 6;13(9):12973–95. doi: 10.18632/aging.202969 (PMC8148504; doi:10.18632/aging.202969)
Supplement: Supplementary Tables 1, 2, 5 [file aging-13-202969-s002.pdf]

SUPPLEMENTARY TABLES

Supplementary Table 1. Summary of Illumina post-sequencing sequence assembly.

Sample	Clean reads	Clean bases	Error rate (%)	Q20 (%)	Q30 (%)	GC content (%)
SCI_KD1	23065121	1.15E+09	0	100	100	48.6
SCI_KD2	23109839	1.16E+09	0	100	100	48.9
SCI_KD3	23240415	1.16E+09	0	100	100	48.55
SCI_KD4	23094364	1.15E+09	0	100	100	49.56
SCI_KD5	23093374	1.15E+09	0	100	100	49.74
SCI_SD1	23705569	1.19E+09	0	100	100	48.98
SCI_SD2	23294618	1.16E+09	0	100	100	48.71
SCI_SD3	22920879	1.15E+09	0	100	100	48.48
Sham_KD1	23720739	1.19E+09	0	100	100	49.27
Sham_KD2	23702903	1.18E+09	0	100	100	49.77
Sham_KD3	23382678	1.17E+09	0	100	100	49.86
Sham_SD1	23486556	1.17E+09	0	100	100	49.97
Sham_SD2	23150638	1.16E+09	0	100	100	48.86
Sham_SD3	23165313	1.16E+09	0	100	100	48.62
Sham_SD4	21452399	1.07E+09	0	100	100	48.96

Supplementary Table 2. Mapping results between the transcriptome and reference genome.

Sample	Total reads	Total mapped	Multiple mapped	Uniquely mapped
SCI_KD1	23065121	21443709 (92.97%)	2274693 (9.86%)	19169016 (83.11%)
SCI_KD2	23109839	21515285 (93.1%)	2212778 (9.58%)	19302507 (83.53%)
SCI_KD3	23240415	21605446 (92.96%)	2322963 (10.0%)	19282483 (82.97%)
SCI_KD4	23094364	21523546 (93.2%)	2150060 (9.31%)	19373486 (83.89%)
SCI_KD5	23093374	21509560 (93.14%)	2239829 (9.7%)	19269731 (83.44%)
SCI_SD1	23705569	22083424 (93.16%)	2566694 (10.83%)	19516730 (82.33%)
SCI_SD2	23294618	21697617 (93.14%)	2301896 (9.88%)	19395721 (83.26%)
SCI_SD3	22920879	21254877 (92.73%)	2395814 (10.45%)	18859063 (82.28%)
Sham_KD1	23720739	22140211 (93.34%)	2187062 (9.22%)	19953149 (84.12%)
Sham_KD2	23702903	22091787 (93.2%)	2239448 (9.45%)	19852339 (83.75%)
Sham_KD3	23382678	21861517 (93.49%)	2109646 (9.02%)	19751871 (84.47%)
Sham_SD1	23486556	21964601 (93.52%)	2094514 (8.92%)	19870087 (84.6%)
Sham_SD2	23150638	21578282 (93.21%)	2172727 (9.39%)	19405555 (83.82%)
Sham_SD3	23165313	21556978 (93.06%)	2303960 (9.95%)	19253018 (83.11%)
Sham_SD4	21452399	19967854 (93.08%)	2080077 (9.7%)	17887777 (83.38%)

Supplementary Table 5. A statistical table of the number of members (genes/transcripts) contained in each module. The first column is the module (indicated by the colour), and the second column is the number of members belonging to the module.

module	number
blue	1221
brown	873
turquoise	1877
grey	716
yellow	263
green	213
